# Effects of matrine on the proliferation of HT29 human colon cancer cells and its antitumor mechanism

**DOI:** 10.3892/ol.2013.1449

**Published:** 2013-07-08

**Authors:** CHENG CHANG, SHAO-PING LIU, CHUN-HUA FANG, REN-SHENG HE, ZHEN WANG, YOU-QING ZHU, SHAO-WEI JIANG

**Affiliations:** 1 Department of Gastroenterology, Huangshi Central Hospital of Hubei Polytechnic University, Huangshi, Hubei 435000, P.R. China; 2 Department of Radiology, Huangshi Central Hospital of Hubei Polytechnic University, Huangshi, Hubei 435000, P.R. China; 3 Hubei Key Laboratory of Kidney Disease Pathogenesis and Intervention, Huangshi Central Hospital of Hubei Polytechnic University, Huangshi, Hubei 435000, P.R. China; 3 Department of Gastroenterology, Zhongnan Hospital of Wuhan University, Wuhan, Hubei 430071, P.R. China; 4 Department of Immunology, Hubei University of Chinese Medicine, Wuhan, Hubei 430065, P.R. China

**Keywords:** matrine, colon cancer HT29 cells, anti-proliferative effects

## Abstract

Matrine is one of the main active components that is extracted from the dry roots of *Sophora flavescens*. The compound has potent antitumor activity in various cancer cell lines. However, the anticancer activity of matrine in colon cancer cells remains unclear. The purpose of the present study was to investigate the effects of matrine on the growth of human colon cancer cells and the expression of the associated proteins. Cancer cell proliferation was measured by 3-(4,5-dimethylthiazolyl)-2,5-diphenyl-tetrazolium bromide (MTT) assay. The cell cycle distribution and apoptosis were analyzed by flow cytometry (FCM). The activation of the caspases and the expression of pro-apoptotic and anti-apoptotic factors were examined using western blot analysis. Matrine was shown to significantly inhibit the proliferation of HT29 cells in a dose- and time-dependent manner, and also to reduce the percentage of cells in the G_2_/M phase, which was most frequently associated with an increase of cells arrested in the G_0_/G_1_ phase of the cell cycle. Western blot analysis revealed that matrine induced the activation of caspase-3 and -9 and the release of cytochrome C (Cyto C) from the mitochondria to the cytosol. Furthermore, the pro-apoptotic factor, Bax, was upregulated and the anti-apoptotic factor, Bcl-2, was downregulated, eventually leading to a reduction in the ratio of Bcl-2/Bax proteins. The results demonstrated that matrine inhibits proliferation and induces apoptosis of HT29 human cells *in vitro*. The induction of apoptosis appears to occur through the upregulation of Bax, the downregulation of Bcl-2, the release of Cyto C from the mitochondria to the cytosol and the activation of caspase-3 and caspase-9, which subsequently trigger major apoptotic cascades. Matrine has potent antitumor activity in HT29 cells and may be used as a novel effective reagent in the treatment of colon cancer.

## Introduction

Colon cancer is the second most prevalent malignancy and the third leading cause of cancer-related mortalities worldwide, resulting in ~500,000 mortalities every year. Colon cancer is highly lethal and aggressively malignant due to its dormant course, difficult early diagnosis, metastasis, strong invasion and poor prognosis (1–4). Surgical resection remains the only curative treatment for colorectal cancer, however, the outcome is not always satisfactory. Although 70–80% of patients are eligible for curative surgical resection at the time of diagnosis, 50% of all newly-diagnosed patients ultimately develop metastatic disease. Numerous patients should be considered for palliative treatment, including chemotherapy and radiotherapy. However, the toxicity of these chemotherapy medicines to normal tissues and cells has been a major obstacle in successful cancer treatment (5–11). There is an urgent requirement to identify novel natural compounds with a low toxicity and high selectivity for killing cancer cells.

Traditional Chinese medicine has been practiced for several millennia and includes a large number of recipes that have not yet been fully explored scientifically. *Sophora flavescens* Ait is an example of one of these unexplored substances. *Sophora flavescens* Ait is a leguminous plant that grows in China, Japan and certain European countries. The dry root of the plant is extensively used in traditional Chinese medicine for the treatment of viral hepatitis, cancer, cardiac diseases and skin diseases, including atopic dermatitis and eczema (12–16).

Matrine is one of the main alkaloid components that may be extracted from the *Sophora* root. The compound, which was first isolated and identified in 1958 from *Sophora flavescens* Ait, has a molecular formula of C_15_H_24_N_2O_. In China, matrine has been widely used in the treatment of various diseases, since it has a wide range of pharmacological effects, including anti-inflammatory, antiviral, immunoinhibitory, antifibrotic, analgesic, antiarrhythmic and anti-diarrheal effects. Interest has been generated in the antitumor activity of matrine. It has been reported that matrine exerts antitumor effects by inhibiting proliferation and inducing the apoptosis of gastric and cervical cancer and leukemia and glioma cells. Matrine has also been shown to induce apoptosis of murine hepatoma cells *in vitro* and *in vivo,* as well as inhibiting tumor growth. Furthermore, matrine inhibits the adhesion and migration of cervical cancer HeLa cells, the invasion and metastasis of human malignant melanoma A375 cells and the growth of established gastric tumors in mice (17–20). However, whether or not matrine is able to inhibit the proliferation of human colon cancer HT29 cells and its molecular mechanisms of action are unclear. Therefore, the present study aimed to investigate the antitumor effect of matrine in human colon cancer HT29 cells, and to further elucidate its molecular mechanism involved in antineoplastic activities.

## Materials and methods

### Reagents and chemicals

**Figure 1:**
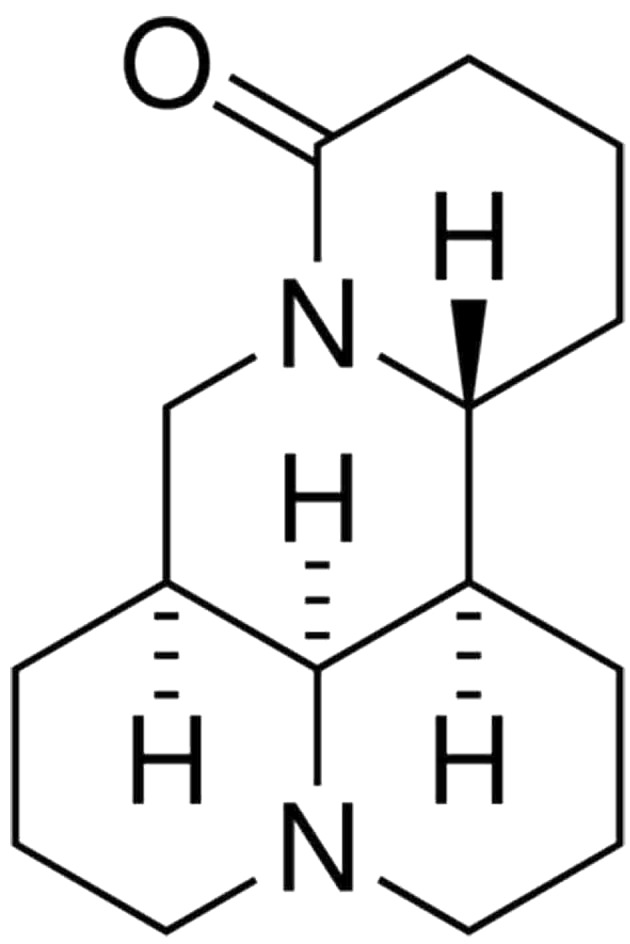
Chemical structure of matrine (C_15_H_24_N_2O_).

Matrine, the chemical structure of which is shown in Fig. 1, was purchased from Sigma-Aldrich (St Louis, MO, USA), with a purity of >98%, as confirmed by high-performance liquid chromatography (HPLC). The molecular formula of matrine is C_15_H_24_N_2O_ and its molecular weight is 248.36. In the present study, matrine was dissolved in cell culture medium at a stock concentration of 20 mg/ml and stored at −20°C. The stock solution was freshly diluted in the medium prior to being used in each experiment. Fetal bovine serum was purchased from Zhejiang Tianhang Biological Technology Co., Ltd. (Hangzhou, China). RPMI-1640 medium was bought from Keygen Biotechnology Co., Ltd. (Nanjing, China). Sodium dodecyl sulfate (SDS), 3-(4,5-dimethylthiazol2-yl)-2,5-diphenyltetrazolium bromide (MTT), L-glutamine and Annexin V fluorescein isothiocyanate/propidium iodide (Annexin V-FITC/PI) apoptosis detection kits were purchased from Beijing Biosea Biotechnology Co., Ltd. (Beijing, China). Antibodies specific for Bcl-2, Bax, cytochrome C (Cyto C) and β-actin were obtained from R&D Systems Inc. (Minneapolis, MN, USA). Anti-caspase-3 and -9 were purchased from Wuhan Boster Bio-engineering Co., Ltd., (Wuhan, China) and the JC-1 probe was from the Beyotime Institute of Biotechnology (Nantong, China).

### Cell line and cell culture

The human colon cancer HT29 cell line was obtained from the Department of Oncology (Zhongnan Hospital of Wuhan University, Wuhan, China). Cells were grown in RPMI-1640 medium supplemented with 10% heat inactivated fetal calf serum (FCS), 1% L-glutamine and 1% penicillin-streptomycin in a humidified atmosphere containing 5% CO_2_. The HT29 cells were grown in a monolayer culture using 25-cm^2^ tissue culture flasks and were periodically detached from the flask surface using 1% trypsin-ethylene-diamine tetraacetic acid (trypsin-EDTA) solution. The cell counts were determined using a CC-108 microcellcounter (Sysmex, Kobe, Japan). Cells in the logarithmic phase of growth were used for all studies described.

### MTT assay

The MTT assay detects the reduction in MTT by mitochondrial dehydrogenase to form a blue formazan product, which reflects the normal functioning of mitochondria and hence the cell viability. Subsequent to being incubated with matrine for 24, 36 or 48 h, in 96-well plates, the cells (10^4^/well) were washed twice with phosphate-buffered saline (PBS) and MTT (100 μg/0.1 ml PBS ) was added to each well. The cells were incubated at 37°C for 4 h. The formazan crystals were dissolved by adding 100 μl DMSO and the absorbance was measured at 570 nm using a spectrophotometer. The cell proliferation inhibition rate was calculated as 1 - (average OD value of the wells with the administered drug/average OD value of the control wells) × 100. The proliferation response of the HT29 cells was determined by the MTT assay as described previously. The experiments and all the subsequent assays were repeated three times.

### Cell cycle analysis

To analyze the cell cycle, the cells were collected in the phase of logarithmic growth and the cell concentration was adjusted to 1×10^6^ cells/ml. The cells were treated with various concentrations of matrine (4, 8 or 16 mg/ml). Following 24 h of treatment, the floating and attached cells were collected and centrifuged, washed with cold PBS and fixed in 70% cold ethanol overnight at 4°C. A fluorochrome solution containing 50 μg/ml PI, 3.4 mmol/l sodium citration, 20 μg/ml RNase A and 1% Triton X-100 was added and the mixture was incubated in the dark at room temperature for 30 min. The distribution of the cell cycle was measured using flow cytometry (FCM; Partec, Münster, Germany). FCM analysis was performed using the Cell Quest software (Beckton Dickinson and Company, Franklin Lakes, NJ, USA).

### Annexin V-FITC/PI double staining for FCM-assessed apoptosis

The Annexin-V-FITC/PI double staining assay was used to detect cellular apoptosis. The HT29 cells were equally distributed into culture flasks and treated with matrine concentrations of 0, 4, 8 and 16 mg/ml, for 24 h. The cells were collected, washed with cold PBS and resuspended at 1×10^6^ cells/ml in Annexin-V binding buffer. The supernatant (100 μl/tube) was incubated with 5 μl Annexin-V-FITC and 5 μl PI for 15 min at room temperature in the dark. Binding buffer (400 μl) was then added to each tube and followed by cytometric analysis within 1 h of staining. All experiments were repeated three times.

### Detection of Cyto C release from the mitochondria to the cytosol

Cyto C determination in cytosolic and mitochondrial fractions was detected using western blot analysis. The cells were harvested following the respective treatments and washed once with ice-cold PBS. In order to isolate the mitochondria and cytosol, the cells were sonicated in buffer containing 10 mM Tris-HCl pH 7.5, 10 mM NaCl, 175 mM sucrose and 12.5 mM EDTA and the cell extract was centrifuged at 1,000 × g for 10 min to pellet the nuclei. The supernatant obtained was centrifuged at 18,000 × g for 30 min to pellet the mitochondria and purified as previously described. The resulting supernatant was termed the cytosolic fraction. The pellet was lysed and the protein content was estimated in the two fractions using Bradford’s method. Equal amounts of protein were separated on 15% SDS-PAGE and electrotransferred to a polyvinylidene fluoride (PVDF) membrane. The membrane was then incubated in 5% skimmed milk in TBST [Tris-buffered saline (TBS) composed of 10 mM Tris, 150 mM NaCl (pH 7.6), with 0.1% Tween 20] for 2 h, followed by overnight incubation with the primary antibody separately. The incubated membranes were extensively washed with TBST prior to incubation for 2 h with the secondary antibody. Subsequent to extensive washing with TBST, the immune complexes were detected by an enhanced chemiluminescence (ECL) detection kit (Amersham Pharmacia Biotech, Piscataway, NJ, USA).

### Western blot analysis

The effects of matrine on protein expression was analyzed using western blot analysis. Following treatment with matrine, the floating and adherent cells were lysed in buffer A [10 mM Tris-HCl (pH 7.6), 1 mM EDTA, 10% glycerol, 1 mg/ml leupeptin, 1 mg/ml pepstatin, 2 μg/ml aprotinin and 3.28 mg/ml PMSF] by three consecutive 10-sec sonications with a Tekmar sonic disrupter (Sigma-Aldrich) at power setting 60. Proteins were separated by SDS-PAGE (5% stacking gel; 10% separating gel) and transferred to nitrocellulose (Micron Separation Inc., Westborough, MA, USA) using a semi-dry blotting apparatus (Bio-Rad, Hercules, CA, USA). The membranes were incubated with 5% skimmed dry milk overnight at room temperature. The membrane was then washed three times with TBST [10 mM Tris-HCl (pH 8.0), 150 mM NaCl and 0.5% Tween-20] for 5 min each, at room temperature and then probed with an appropriate titer of antibody (Cell Signaling, Beverly, MA, USA) that binds to Bcl-2, Bax, caspase- 3 or -9 or β-actin, for 1 h at room temperature. Subsequently, the membrane was washed as described previously and further incubated with horseradish peroxidase-conjugated sheep anti-mouse IgG (monoclonal primary antibodies) or goat anti-rabbit IgG (poly-clonal primary antibodies; Amersham Pharmacia Biotech) at room temperature for 1 h. The membrane was washed three times with TBST and developed using the ECL kit (Amersham Pharmacia Biotech). The intensity of the immunoreactive bands was quantified using a densitometer (Molecular Dynamics, Sunnyvale, CA, USA).

### Statistical analysis

The results were expressed as the mean ± standard deviation (SD). An analysis of variance (ANOVA) and Dunnett t-test were used to evaluate the statistical significance. P<0.05 was considered to indicate a statistically significant difference.

## Results

### Inhibitory effect of matrine on the growth of HT29 cells

**Figure 2:**
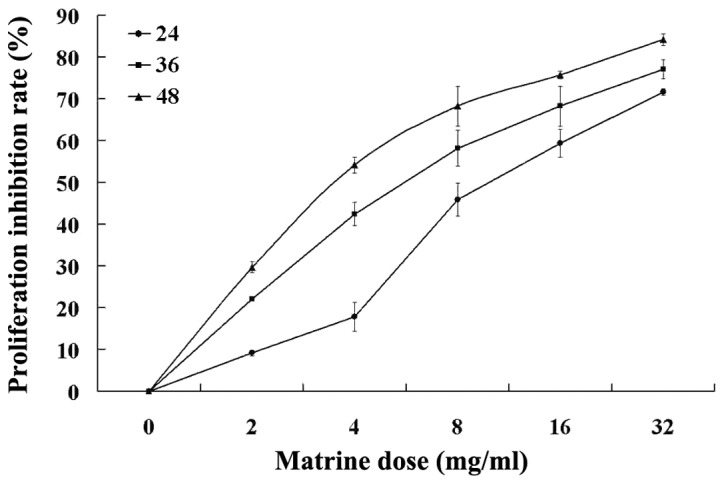
Anti-proliferative effect of matrine. As the concentration increased, the growth of HT29 cells was markedly inhibited in a time- and dose-dependent manner.

The anti-proliferative effect of matrine on the HT29 cells was detected by MTT assay. The results show that as concentration increased, the proliferation of the HT29 cells was markedly inhibited in a dose- and time-dependent manner by matrine concentrations of 2–32 mg/ml for 24, 36 and 48 h *in vitro* (P<0.05; Table I and Fig. 2).

### Matrine-induced G_0_/G_1_ cell cycle arrest

**Figure 3:**
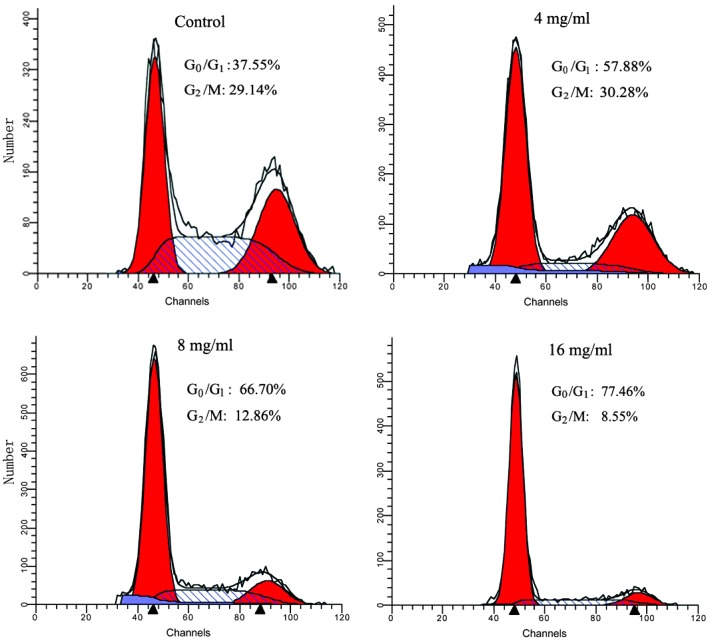
Cell cycle analysis. Representative DNA fluorescence histograms of PI stained cells. The HT29 cells were untreated (control) or treated with 4, 8 or 16 mg/ml matrine, with 37.55, 57.88, 66.70 and 77.46% G_0_/G_1_ cells, respectively, as assessed by flow cytometry (FCM). PI, propidium idiode.

To examine the effect of matrine on cell cycle progression, the cells that were untreated or treated with matrine for the indicated concentrations (4, 8 or 16 mg/ml) and the cell cycle distribution were analyzed using FCM. As shown in Fig. 3, matrine significantly increased the number of cells in the G_0_/G_1_ phase and decreased the number of cells in the G_2_/M phase in a dose-dependent manner, indicating that matrine caused a growth arrest of HT29 cells in the G_0_/G_1_ cell cycle phase, leading to a depletion of S and G_2_/M phase cells.

### Detection of apoptosis

**Figure 4:**
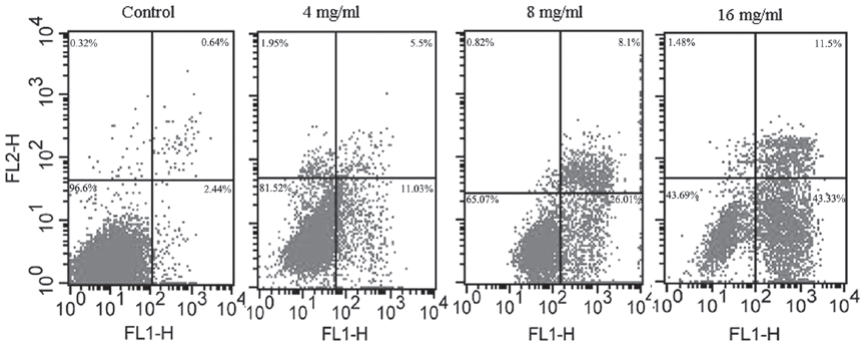
Matrine-induced apoptosis. HT29 cells were treated without (control) or with matrine at concentrations of 4, 8 or 16 mg/ml for 24 h, then processed for Annexin V/propidium iodide (PI) staining and analyzed using flow cytometry (FCM). The viable cells are located in the lower left corner (negative in Annexin V-FITC and PI). Early apoptotic cells are in the lower right corner (Annexin V-FITC positive). Late apoptotic cells showing signs of progressive cellular membrane and nuclear damage are in the upper right corner (double positive). FL1-H, Annexin V-FITC; FL2-H, PI.

Perturbations in the cell membrane occur during the early stages of apoptosis and lead to a redistribution of phosphatidylserine to the external side of the cell membrane. Annexin V selectively binds to phosphatidylserine and thus enables the use of a fluorescein-labeled Annexin V kit to identify the cells that are undergoing apoptosis (21). An Annexin-V-FITC/PI double staining assay was performed to detect the apoptosis of the HT29 cells. The cells were also stained with PI to distinguish early apoptotic cells from necrotic cells. A FACScan flow cytometer collected 10,000 events. The percentage of live, dead and apoptotic cells was determined as described in the methods and shown in Fig. 4. The viable cells are located in the lower left corner (negative in Annexin V-FITC and PI). Early apoptotic cells are in the lower right corner (Annexin V-FITC positive). Late apoptotic cells showing signs of progressive cellular membrane and nuclear damage are in the upper right corner (double positive).

### Cyto C release from mitochondria to the cytosol

**Figure 5:**
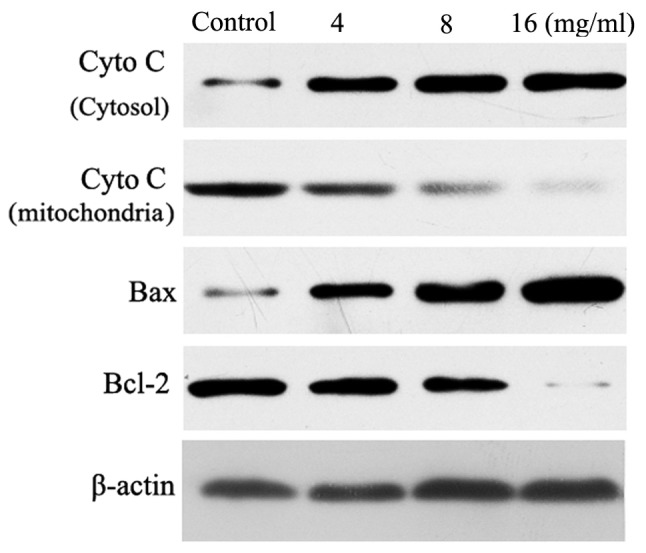
Effect of matrine on the expression of Cyto C, Bax and Bcl-2 proteins in the HT29 cells. Cyto C, cytochrome C.

In response to certain apoptotic stimuli, Cyto C is released from the mitochondria to the cytoplasm, where it combines with Apaf-1 to promote apoptosome assembly and caspase-9 activation. The release of Cyto C from the mitochondria is a critical step in the apoptotic cascade, since this activates downstream caspases. Therefore, western blotting was performed in the cytosolic and mitochondrial fractions to investigate the release of Cyto C in the matrine-treated HT29 cells. As shown in Fig. 5, the results demonstrate a concentration-dependent increase in the cytosolic Cyto C levels following treatment with matrine. Simultaneously, a decrease in Cyto C was observed in the mitochondrial fraction.

### Expression of Bax and Bcl-2 protein

To understand the anti-proliferative mechanisms of matrine in human colon cancer HT29 cells, the expression of apoptotic-related proteins were investigated (Fig. 5). Western blot analysis confirmed that matrine at concentrations of 4–16 mg/ml dose-dependently downregulated Bcl-2 protein and upregulated Bax protein, eventually leading to a reduction in the ratio of Bcl-2/Bax protein. Studies have shown that Bcl-2 and its dominant inhibitor, Bax, are key regulators of cell proliferation and apoptosis (22,23). The overexpression of Bcl-2 enhances cell survival by suppressing apoptosis, but the overexpression of Bax accelerates cell death. The induction of apoptosis, cell cycle arrest and a decrease in the ratios of Bcl-2/Bax protein caused by matrine may be significant matrine anti-proliferative mechanisms against cancer cells.

### Activation of caspase-9 and -3

**Figure 6:**
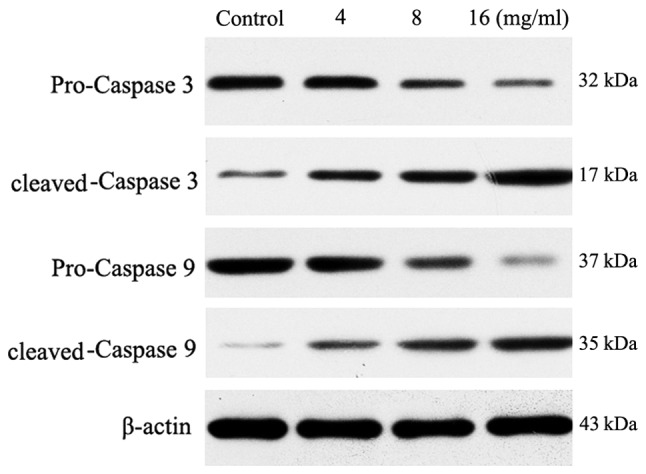
Caspase-3 and -9 expression in the matrine-treated HT29 cells.

Western blot analysis of cleaved and full length caspase-9 revealed that matrine induced a gradual increase in caspase-9 processing with various matrine concentrations at 4, 8 or 16 mg/ml for 24 h following treatment (Fig. 6). The amount of cleaved caspase-9 fragments then reached a plateau at the dose of 16 mg/ml. The appearance of processed fragments was accompanied by a decrease in the full-length enzyme. No processed fragments were detected in the control extracts. Processing of caspase-3, an effecter caspase that is directly activated by caspase-9, was also examined. The 17 kDa cleaved caspase-3 fragments were detected at 24 h following matrine treatment and an additional increase in the amount of fragments was observed.

## Discussion

Cancer chemoprevention is defined as inhibiting, delaying or reversing the carcinogenic process using non-toxic chemicals, and is considered to be a promising strategy for controlling cancer progression. A variety of chemical compounds have been reported to protect against chemical carcinogenesis and thus, are considered to be cancer chemopreventive agents. Among these, matrine is a promising phytochemical agent that has attracted interest due to its cancer chemopreventive activity in multistage carcinogenesis. Matrine is a naturally occurring polyphenolic phytoalexin, which has been demonstrated to display cancer chemopreventive activity in *in vivo* animal experiments. It was first isolated and identified in 1958 from *Sophora flavescens* Ait and has been widely used in China as a therapeutic agent against cancer. Matrine has been considered as a good and convenient Chinese herbal preparation with low toxicity and few side-effects. It has also been administered to children and infants. However, to the best of our knowledge, the effect of matrine on pancreatic cancer has not been previously reported (24,25).

Uncontrolled proliferation is a significant biological feature of cancer cells, and inhibiting cell proliferation may achieve the arrest of tumor growth (17,26). The present study investigated whether matrine was able to reduce the proliferation rate of tumor cells. As shown in the MTT assay, the growth of the HT29 cells was inhibited in a dose- and time-dependent manner when treated with 4–16 mg/ml matrine. FCM revealed that matrine markedly arrested the HT29 cells in the G_0_/G_1_ phase of the cell cycle (Fig. 3), indicating that retardation of cell cycle progression may be a mechanism that underlies the anti-proliferative effect of matrine.

Apoptosis is not only a significant phenomenon in normal cells, but it is also associated with the development of numerous diseases. An imbalance of apoptosis and proliferation is a significant cause for the development and progression of a tumor. The inhibition of proliferation and the induction of apoptosis in tumor cells are main treatment strategies in combined anticancer therapy (27–33) To determine the anticancer mechanisms of matrine, the pro-apoptotic properties of the compound were assessed in the present study. The results from the Annexin V-FITC/PI double staining suggested that matrine was able to induce apoptosis in a dose-dependent manner in the range of 4–16 mg/ml. The results of the present study suggest that matrine, by a reduction in tumor cell proliferation combined with an induction of tumor cell apoptosis, may be useful for the two aspects of the anticancer treatment strategy.

Since the mechanisms through which these compounds induce cell death are not completely understood, the changes in the expression and localization of several apoptosis-related proteins were examined in HT29 cells in the present study.

The Bcl-2 family of proteins, including Bcl-2 and Bax, function to control cell proliferation, differentiation and programmed cell death, and consist of pro- and anti-apoptotic family members. One of the main regulatory steps of apoptotic cell death is controlled by the ratio of anti- and pro-apoptotic members of the Bcl-2 family of proteins, which determines the susceptibility to apoptosis. Bax, a pro-apoptotic factor of the Bcl-2 family, is located in a monomeric form in the cytosol or loosely attached to the membranes under normal conditions. Following a death stimulus, cytosolic and monomeric Bax translocate to the mitochondria, where they become integral membrane proteins, which are able to cross-link as homodimers, allowing for the release of factors from the mitochondria, including Cyto C, to propagate the apoptotic pathway. Bcl-2 and its associated proteins control the release of Cyto C from the mitochondria. Hallmarks of the apoptotic process include the activation of cysteine proteases, which represent initiators and executors of cell death. In the cytosol, Cyto C activates caspase-9, which in turn activates effector caspases, including caspase-3 (34–37). In the present study, matrine reduced the anti-apoptotic/pro-apoptotic Bcl-2/Bax ratio. In addition to this, matrine caused the release of Cyto C from the mitochondria and subsequently increased caspase-3 activity. Caspase-3 is synthesized as a 32-kDa inactive precursor, which is proteolytically cleaved to produce a mature enzyme composed of 17-kDa fragments. In the present study, the procaspase-3 protein was reduced and the caspase-3 in a cleaved form was increased in the apoptotic HT29 cells following treatment with matrine at concentrations of 4–16 mg/ml (38,39).

In summary, the present results demonstrated that matrine was able to suppress HT29 cell proliferation and induce apoptosis and cell cycle arrest at the G_0_/G_1_ phase by targeting the Cyto C, caspase-9, caspase-3, Bcl-2 and Bax mitochondrial apoptotic pathway. These findings suggest that matrine may have wide therapeutic and/or adjuvant therapeutic application in the treatment of human colon cancer.

## Figures and Tables

**Table I:** Various concentrations of matrine inhibit the proliferation of HT29 cells.

Concentration (ml/l)	24 h	36 h	48 h
Control	0.00±0.07	0.00±0.11	0.00±0.14
2	9.20±0.65^a^	22.06±0.20^a^	29.73±1.29^a^
4	17.88±3.51^a^	42.48±2.78^a^	54.22±1.91^a^
8	45.96±3.95^b^	58.20±4.33^b^	68.29±4.79^b^
16	59.35±3.36^b^	68.29±4.79^b^	75.75±0.92^b^
32	71.62±0.76^b^	77.14±2.30^b^	84.20±1.47^b^

a P<0.05 vs. control.

b P<0.01 vs. control.

All data are presented as mean ± standard deviation (SD).
